# Comparison of the healing process of xenografts with three different sources in critical-size bone defects: An in vivo study

**DOI:** 10.34172/japid.2024.004

**Published:** 2024-03-16

**Authors:** Reza Amid, Mahdi Kadkhodazadeh, Aida Kheiri, Shiva Esfandiari

**Affiliations:** ^1^Dental Research Center, Research Institute of Dental Sciences, School of Dentistry, Shahid Beheshti University of Medical Sciences, Tehran, Iran; ^2^Department of Periodontics, School of Dentistry, Shahid Beheshti University of Medical Sciences, Tehran, Iran; ^3^Department of Biology, School of Science, Shahid Beheshti University, Tehran, Iran

**Keywords:** Bone grafting, Bone regeneration, Bone substitutes, Xenograft

## Abstract

**Background.:**

Xenograft bone substitutes can be obtained from different animals and processed using various methods. The present in vivo study evaluated bone regeneration after using three types of xenografts with different sources in critical-sized bone defects in rabbit calvaria.

**Methods.:**

Four 8-mm defects were created in calvaria of 14 New Zealand and white male rabbits. Three out of four defects were filled with xenografts of bovine, camel, and ostrich sources. The fourth defect was left unfilled as the control group. Seven rabbits were sacrificed after eight weeks and seven others after 12 weeks. Micro-CT imaging and histologic evaluation were further performed on dissected calvarias.

**Results.:**

After 8 and 12 weeks, the highest and lowest percentages of new bone formation were observed in the camel (27.71% and 41.92%) and control (11.33% and 15.96%) groups, respectively. In the case of residual material, the ostrich group had the most value after eight weeks (53%), while after 12 weeks, it was highest in the camel group (37%). Micro-CT findings were consistent with histologic results.

**Conclusion.:**

Although all three xenografts can be good choices for treating bone defects, camel-sourced xenograft seemed to be better than the other two groups. The origin and processing procedures of xenografts affected their final characteristics, which should be considered for clinical use.

## Introduction

 Bone loss following trauma, cancer, tooth extraction, and bone diseases is an irreversible process.^1^ Various methods, such as distraction osteogenesis, inlay/onlay bone grafting, and guided bone regeneration, have been proposed to reconstruct critical-size bone defects (CSDs), which cannot be healed spontaneously.^2,3^ Bone tissue engineering is an almost new procedure. Approximately 2.2 million bone grafting procedures are performed yearly to treat bone defects, making this treatment the second most common operation globally.^1,4^ This technique consists of a triad of multipotent progenitor stem cells, scaffolds, and signaling molecules. It appears to be a reliable method to treat and fill osseous defects by stimulating bone remodeling and replacing the missing bone.^5-8^

 An acceptable bone graft should have specific properties, including biocompatibility, high availability, osteoconductivity, proper structural support, and biomechanical properties similar to the bones at the implant site.^3,8-10^ Up to now, different biomaterials have been introduced as bone replacements.^11^ Autologous bone graft has been considered the gold standard as it keeps osteogenic cells and proteins and ensures a limited possibility of rejection.^12-14^ However, patient morbidity, additional surgery, and limited graft availability have decreased its use.^2,12,13^ Allograft, another alternative, is available in different sizes and has resolved some of the drawbacks. However, it has some disadvantages, including the risk of disease transmission, rapid resorption, donor limitation, and immune response.^4,15-17^

 Xenograft is another type of bone graft derived from different species like cow, ostrich, pig, and camel. These bone grafts have osteoconductive properties and do not cause immune responses.^18^ Biocompatibility, porous structure, appropriate osteoconductive properties, affordable costs, and being rich in sources can be some of the advantages of xenografts.^2,8,11^ Xenografts are prepared using different methods, including heat treatment, hydrothermal hydrolysis by NaOH, ethylenediamine, or sodium hypochlorite. A key point during these procedures is to remove organic matter, xenogeneic antigens, and cells while keeping the natural biological features.^8,16^ Proper scaffolds must have ideal porosity and pore size with a well-interconnected pore system. Moreover, particle size and Ca/P ratio are also of great importance. All these features would lead to cell adhesion, proliferation and migration of cells, drug release, and vascularization.^2,3,12^

 Because of the differences in xenograft sources and preparation methods, xenograft bone substitutes may represent a variety of behaviors in clinical use.^2,13^ Moreover, no study has compared the ability of xenografts with different sources in bone healing in CSDs. Therefore, the present in vivo study compared and evaluated bone regeneration of CSDs in rabbit calvaria after using three types of xenografts with different sources.

## Methods

###  Animal model 

 Rabbits were selected for this study since it has been demonstrated to be isomorphic to clinical situations and represent high bone turnover, ease of handling, and the possibility of creating multiple defects.^19,20^ Fourteen New Zealand and white male rabbits, with an average age of 12 months, weighing about 2.5‒3 kg, were used. Each rabbit was acclimatized for 14 days in an individual cage (60*45*52 cm), had a diet of commercial pellets (BehParvar Co., Iran), and had access to water ad libitum.

###  Surgical procedure

**Figure 1:**
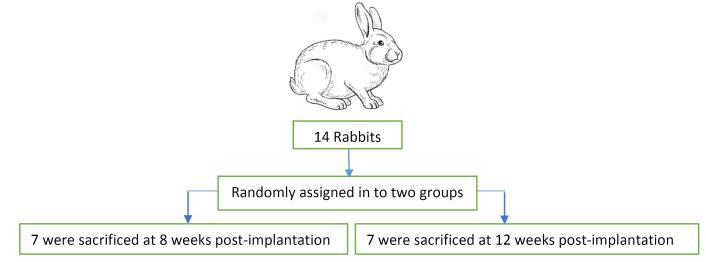
Grouping 14 rabbits in the study

 All the surgical procedures were conducted by an expert blinded surgeon. The animals were randomly divided into two equal groups (8-week- and 12-week) (Figure 1).^18^ At the time of surgery, the animals were anesthetized by intramuscular (IM) injection of a combination of ketamine (35 mg/kg) and 2% xylazine hydrochloride (2 mg/kg). Anesthesia was maintained with isoflurane/nitrous oxide (1:1.5%) and oxygen (2/3:1/3) with a facemask. After complete unconsciousness, trichotomy and asepsis with 10% povidone-iodine were performed on the frontoparietal skin of each animal. The surgical area was also isolated. A single straight incision was made from the nasofrontal suture to the external occipital protuberance.

**Figure 2:**
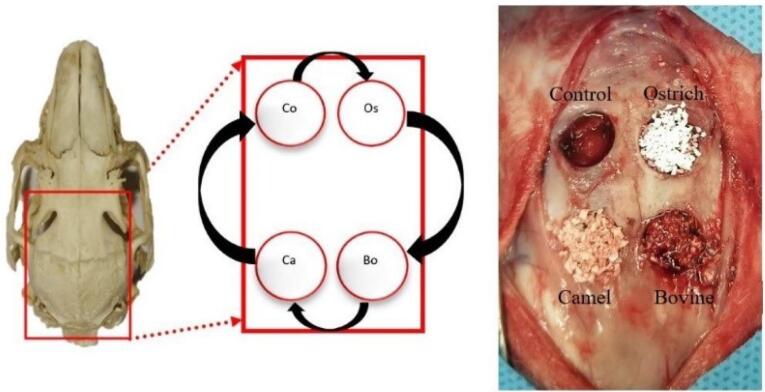
Location of defects and the rotation of filling materials in each sample (Bo: Bovine (OCS-B), Ca: Camel (DCB), Os: Ostrich (DOB), Co: Control)

 Using a periosteal elevator, the flaps were elevated to expose the parietal bone, which was kept open with two hemostats. In each rabbit, four 8-mm round defects were created in the parietal bone with a trephine bur connected to a low-speed handpiece. This procedure was performed under continuous irrigation with 0.9% sterile saline. All the defects were located posterior to the coronal suture and at least 2 mm from the sagittal suture. The round bones were removed completely using a chisel. Care was taken not to damage the dura mater. According to the study design, in each animal, three of the four defects were filled with three different xenografts. Grafts had sources of bovine (OCS-B, NIBEC), camel [Bone PLUS C, NovaTeb holding, (DCB)], and ostrich (Bone PLUS O, NovaTeb Holding, (DOB)]. The fourth defect was left unfilled as the control group. In both the 8-week and 12-week groups, the placement of xenografts in defects was changed on a clockwise rotation (Figure 2).

 Then, the soft tissue was repositioned, and the skin was closed with sutures to achieve primary closure. Following suturing, oxytetracycline antibacterial skin spray was utilized on all surgical wounds. Postoperative care included IM injection of penicillin-sodium antibiotic (50 mg/kg/d) for three days. Each animal was kept in a separate cage and fed sufficient food and water ad libitum. Seven rabbits were sacrificed after eight weeks, and the remaining seven rats were sacrificed after 12 weeks by placing them in a closed jar with lethal doses of ether. Then, the calvariae of the rabbits were dissected.

###  Imaging evaluation

 Calvaria samples were kept in a 10% buffered formalin solution and sent for micro-computed tomography (micro-CT) analysis. The samples were scanned with in vivo x-ray micro-CT scanner (LOTUS inVivo, Behin Negareh Co., Tehran, Iran). All the protocol settings were controlled by LOTUS-inVivo-ACQ software. The total scan time was 20 minutes per sample (1 second per projection, 1200 seconds in total). After placing the samples in a specific holder, the x-ray tube started to rotate 360^o^. Data were collected at 0.01 mA and 80 kVp with less than 5 µm focal spot size and a field of view of 78. The acquired 3D data was reconstructed using LOTUS-inVivo REC by a standard Feldkamp, Davis, Kress (FDK) algorithm. Smoothing filters were adjusted to optimum values for each sample. Afterward, bone volume/tissue volume ratio (BV/TV) was measured using LOTUS-inVivo analysis software.

###  Histologic and histomorphometric analysis

 On average, two central sections of each specimen were used. The sections were fixed in a 10% buffered formalin solution with pH = 7 and decalcified in 10% formic acid for 20 days. Following dehydration with graded alcohol, the samples were embedded in paraffin. From the greatest diameter of the circle, a serial section of at least three cuts with 4 µm of diameter was performed. The sections were stained with hematoxylin and eosin (H&E). A masked pathologist assessed the samples using a light microscope with the objective lens set at × 40, × 100 and × 400.

 For histomorphometric analysis, photographs from four sections of each sample were taken using a camera (Nikon, E8500, Japan) with enough attention to include the entire defect borders. Computer-assisted histomorphometric measurements of new bone formation were obtained using an automated image analysis software (IHMMA, Ver. 1.1, SBMU, Iran). The percentile ratio of the newly formed bone area over the total defect area, residual material, type of bone, and percentage of inflammation (score 0: < 10%, score 1: 10‒30%, score 2: 30‒50%, and score 3: > 50%)^21^ and foreign body reaction were also assessed by the pathologist.

###  Statistical analysis 

 The quantitative data were analyzed using SPSS 24. The Kruskal-Wallis test and two-way ANOVA were used to compare the groups concerning each variable. *P* < 0.05 was considered statistically significant.

## Results

###  Clinical observation 

 All the 14 rabbits were in good health. All the surgical sites were healed without complications.

###  Statistical findings 

 The normality assumption of data in subgroups was checked by the Kolmogorov-Smirnov test, and due to equal sample sizes in each group, all *P* values were > 0.05. Therefore, a slight deviation from the equality of variances was not critical.

###  Histologic findings

####  Type of newly formed bone 

 In the 8-week group, 75% of defects had lamellar bone, and 18% had woven bone. Only 7% of defects showed both lamellar and woven types. In the 12-week group, 53% and 29% of defects had lamellar and woven bone, respectively, and 18% presented both types of bone.

####  Foreign body reaction 

 For the 8-week group, foreign body reaction was observed in 23 defects (out of 28 in total), while in the 12-week group, 20 defects showed this response.

####  Location of bone formation 

 For 60% of defects, osteogenesis happened from the margins of defects to the center. The rest showed bone formation from both their margins and centers.

####  Inflammation 

 Most defects in both groups had a score of 0 ( < 10% of inflammation). Only two defects in the 8-week group filled with DOB showed a score of 3 for inflammation. None of the samples showed signs of necrosis.

###  Histomorphometric findings 

 To compare the regeneration ability of these three different xenografts, the following parameters were assessed in percentage: new bone formation and residual material. In addition, both the mean and 95% confidence interval of each parameter were reported for all subgroups.

####  New bone formation (NBF) 

**Figure 3:**
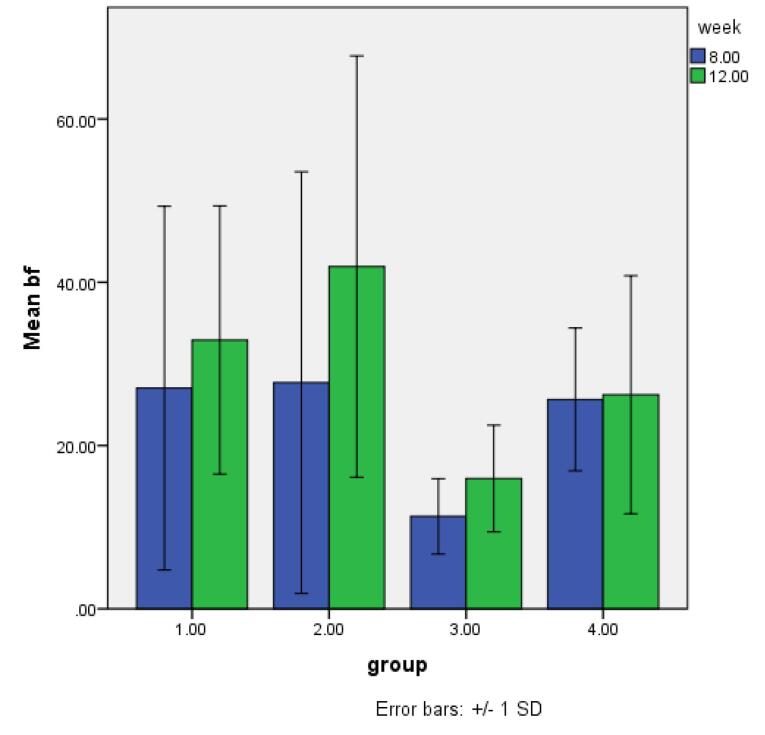
New bone formation in calvarial bone defects, 8 and 12 weeks after implantation with OCS-B, DOB, and DCB (Mean bf: Mean bone formation. 1: OCS-B group, 2: DCB group, 3: Control group, 4: DOB group)

**Figure 4:**
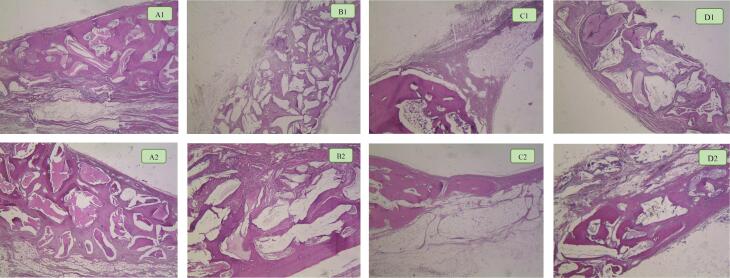
Bone formation in groups after 8 and 12 weeks. A1: OCS-B group after 8 weeks. A2: OCS-B group after 12 weeks. B1: DCB group after 8 weeks. B2: DCB group after 12 weeks. C1: Control group after 8 weeks. C2: Control group after 12 weeks. D1: DOB group after 8 weeks. D2: DOB group after 12 weeks

Figure 3 shows the means for 8-week and 12-week post-implantation subgroups. In both 8-week and 12-week groups, the lowest and highest percentages of NBF were recorded in the control and DCB groups, respectively. Also, the control group had a significant difference from the DCB group after 8 and 12 weeks (*P* < 0.05). Figure 4 shows histological sections of all 4 groups after 8 and 12 weeks.

####  Residual material 

 The quantitative evaluation of residual material at 8 weeks showed that this amount in the DOB group, with 53%, was much higher than that of other groups. After 12 weeks, this percentage was higher in DCB groups with 37% of residual material. More details regarding the residual material percentage of each group are shown in Table 1.

**Table 1:** Mean, standard deviation, and confidence intervals of residual material in the test groups after 8 and 12 weeks

**Groups**	**Mean of RM (%)**	**SD**	**95% CI**
Bovine (OCS-B), 8 weeks	33.33	16.73	20.94, 45.73
Camel (DCB), 8 weeks	42.9	14.48	28.71, 58.09
Ostrich (DOB), 8 weeks	53.02	11.27	43.14, 62.9
Bovine (OCS-B), 12 weeks	30.6	7.92	24.78, 36.52
Camel (DCB), 12 weeks	37.4	7.81	31.15, 43.66
Ostrich (DOB), 8 weeks	33.56	8.57	26.04, 41.08

###  Imaging findings 

####  Mean BV/TV 

**Figure 5:**
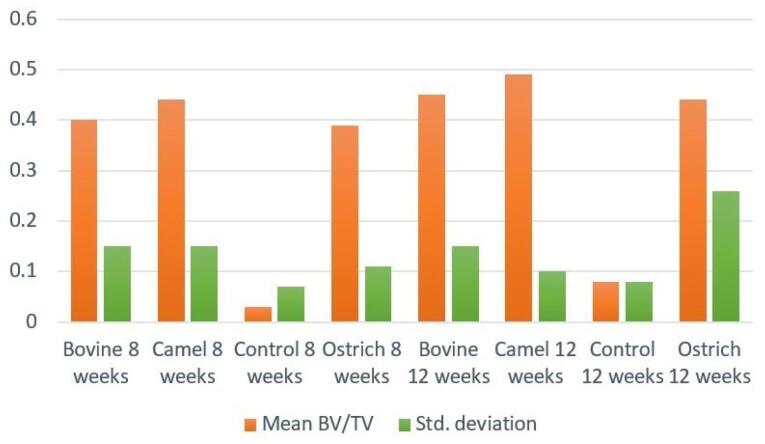
Mean amount of BV/TV in subgroups 8 weeks and 12 weeks after implantation

**Figure 6:**
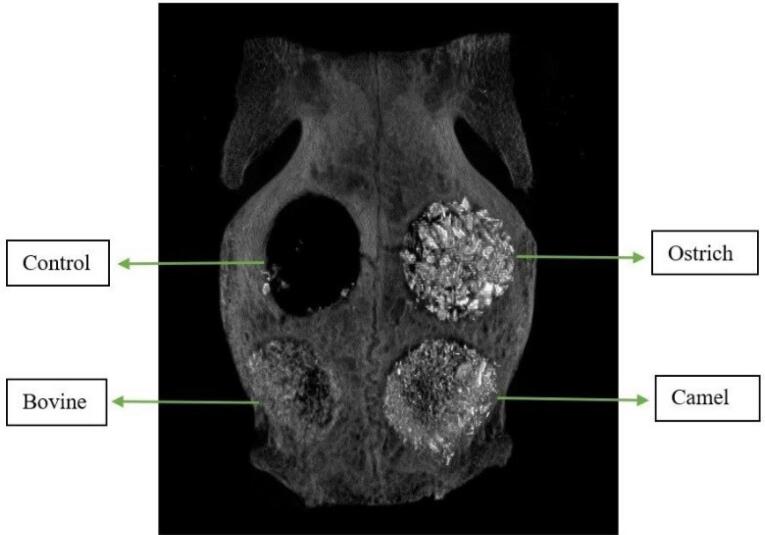
Sample of Micro-CT image of rabbit calvaria filled with three xenografts

Figure 5 shows the means of BV/TV. The highest BV/TV was observed in DCB groups after 8 and 12 weeks, while the lowest amount belonged to the control groups. For both 8-week and 12-week groups, control groups showed significant differences from the other three groups (*P* < 0.001). Figure 6 shows an example of a micro-CT image.

## Discussion

 Although autogenous bone grafts are still considered gold standard grafts for repairing bone defects, several complications such as insufficient supply, donor site morbidity, and high resorption rate have increased the need for an appropriate replacement.^2,11,22^ Xenograft bone substitutes have captured the attention because of the similarity of their inorganic type to deproteinized human bone from porous structure and composition aspects. Xenografts can be made from different sources, but bovine is the first priority in dental treatments.^23,24^ In this study, three deproteinized bone grafts derived from camel, ostrich, and bovine were used to fill the defects in rabbit calvaria to evaluate the effect of xenografts’ origin and properties on bone regeneration ability and newly formed bone’s characteristics.

###  New bone formation 

 Multiple contributing factors, including differences in origin, preparation method, particle size and morphology, inter- and intra-porosity, and an interconnected pore system, might influence vascularization, cell adhesion, diffusion of drugs, osteogenesis, etc. In this study, mean NBF was higher in the DCB group after 8 and 12 weeks, which was significantly different from the control group (*P* < 0.05). As mentioned above, bovine origin was the first to make xenograft bone substitutes, and it is still on top in the global market. Based on our observations, although the difference between the mean NBF of the three study groups was not significant, it seemed that camel was a better origin compared to bovine and ostrich. However, in Ghashtasbi’s^4^ study, after 4 and 8 weeks, OCS-B showed a higher percentage of newly formed bone. In another study by Kiany Yazdi et al,^25^ autogenous bone grafts were compared with one type of xenograft, one allograft, one alloplast, and a gelatin sponge in CSDs of pig calvaria. The results showed that the highest bone formation belonged to the autogenous group, followed by Biomatlante, Bio-Oss®, Exfuse, Stypro gelatin sponge, and control groups.

###  Xenograft properties 

**Figure 7:**
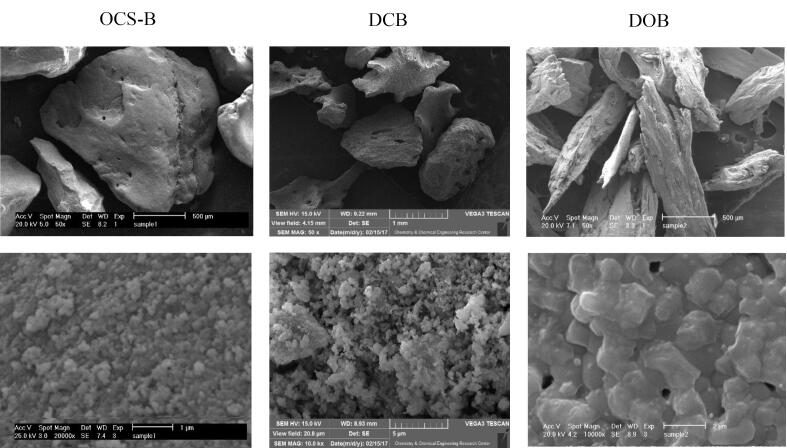
SEM images of OCS-B (left column), deproteinized camel bone (DCB) (middle column), and deproteinized ostrich bone (DOB) (right column) at × 50 and × 10 000 magnifications

 According to manufacturers’ claims, all the three xenografts were prepared using heat treatment with different temperatures. For OCS-B xenograft, 600 ºC was applied,^26^ while for DCB and DOB, 900 ºC has been mentioned. The xenografts used in this study had particle sizes ranging from 250 to 1000 µm, but there was a variety in the morphology of particles in scanning electron microscopy (SEM) assessment (Figure 7), which could have a significant effect on the osteoconductive ability of xenografts. Using × 50 magnification, the variation in shape was presented. OCS-B and DCB showed polyhedral granules, while DOB had spindle-like granules. At × 10 000 magnification, the difference in granules’ surface could be assessed more precisely. OCS-B had a spheroid-like crystal structure, while DCB and DOB had a rod-like one. Although previous reports have mentioned no effects of roughness on osteoblast function,^27^ Block’s^26^ study indicated that the more the particle size was similar to native bone, the better the xenograft could perform its osteoconductivity function.

 Surface area is another important physical characteristic because it is considered a bed for cell adhesion, better metabolic function, and further matrix formation. Based on manufacturers’ claims, the surface area of three xenografts used in this study was as follows: OCS-B (45.109 m^2^/g), DCB (23.817 m^2^/g), and DOB (5.676 m^2^g). The smallest surface area belonged to DOB, which would decrease cell adhesion, angiogenesis, and bone formation. This might explain the lowest amount of bone formation in defects filled with DOB compared to OCS-B and DCB groups after 8 and 12 weeks.

###  Foreign body reaction 

**Figure 8:**
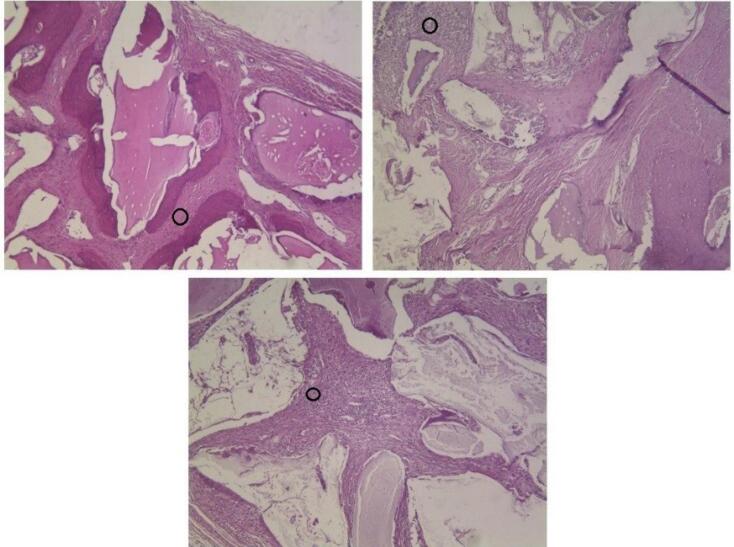
Black circles show aggregation of giant cells around different xenografts

 The majority of defects showed foreign body reactions after 8 and 12 weeks. Foreign body reaction is induced by special cells called multi-nucleated giant cells (MNGCs) that result from the fusion of macrophages. Despite older statements, recent studies indicated that the presence of MNGCs around bone materials was a favorable finding because these cells were capable enough to express growth factors and angiogenic cytokines such as vascular endothelial growth factor (VEGF) and further stimulate bone formation and tissue healing.^28,29^ Based on these reports, the presence of MNGCs in the present study could be a positive finding and may have induced osteoblastic function and further osteogenesis. Figure 8 shows the presence of MNGCs around xenografts in different groups.

###  Residual material 

 After 8 weeks, the DOB group had the highest amount of mean residual material with 53%, which was significantly different from the OCS-B group (*P* < 0.05). The DCB group, with 37% of mean residual material, was the largest after 12 weeks. From 8 weeks to 12 weeks, the DOB group showed a 20% decrease in residual material that was greater than the same parameter in the OCS-B and DCB groups. This decrease could be attributed not only to the preparation process and temperature but also to foreign body reaction and inflammation because score 3 of inflammation was only observed in the DOB group. These factors might lead to a higher possibility of resorption rate in DOB groups. On the other hand, the mean NBF increased only 1% between 8 and 12 weeks. This finding suggests that probably in the DOB group, the main part of osteogenesis had occurred before 8 weeks. Hence, adding a 4-week group will be useful in future studies.

 Takauti et al^30^ used Bio-Oss®, Endobone® xenograft, and an alloplastic to evaluate bone healing in rabbit calvaria. After 8 weeks, xenografts had a higher amount of residual graft, indicating a slower resorption rate of these grafts compared to the alloplast.

###  Micro-CT analysis 

 Micro-CT in vivo studies is a newly emerged method to evaluate bone formation and compare the results with histological findings. In this study, we used micro-CT imaging in all defects after 8 and 12 weeks. Analysis of outcomes presented the proportion of newly formed bone (BV) to the defect volume (TV). This was a good indicator since comparing this parameter between groups was reliable. In our study, the largest and lowest mean BV/TV amount belonged to the DCB and control groups, respectively. This finding was consistent with histological results. The conforming values can increase the validity of histological results and so would be counted as a useful assessment method.

## Limitations

 This study is not without limitations. It is an in vivo study on rabbits, and the results cannot be generalized to humans or other animal species. To more precisely evaluate different bone substitutes, further clinical studies on a larger population and multiple groups of study are needed to assess xenografts’ function during different periods of healing time.

## Conclusion

 According to the results, all three xenografts could promote osteogenesis in CSDs. However, camel seemed to be a better choice of origin as it had higher NBF values than bovine and ostrich. Not only can multiple factors affect xenografts’ properties, but camel and ostrich-derived xenografts are also novel compared to bovine-derived ones. Hence, future investigations are needed to properly select a xenograft in clinical use according to the size and type of defects and the expected healing time.

## Acknowledgments

 The authors wish to thank Dr. Sadegh Hasannia for sharing SEM pictures.

## Competing Interests

 The authors declare no competing interests.

## Consent for Publication

 Not applicable.

## Data Availability Statement

 All data regarding the methodology of the manuscript have been shared.

## Ethical Approval

 The detailed study design was analyzed and approved by the Ethics Committee of the Dental School, Shahid Beheshti University of Medical Sciences, under approval number IR.SBMU.DRC.REC.1398.104.

## Funding

 This research received no specific grant from funding agencies in the public, commercial, or not-for-profit sectors.
